# Quality of life and disease understanding: impact of attending a patient-centered cancer symposium

**DOI:** 10.1002/cam4.422

**Published:** 2015-01-30

**Authors:** Leslie Padrnos, Amylou C Dueck, Robyn Scherber, Pamela Glassley, Rachel Stigge, Donald Northfelt, Joseph Mikhael, Annette Aguirre, Robert M Bennett, Ruben A Mesa

**Affiliations:** 1 Internal Medicine Residency Program, Mayo ClinicScottsdale, Arizona; 2 Division of Health Sciences Research, Mayo ClinicScottsdale, Arizona; 3 Division of Hematology and Medical Oncology, Mayo ClinicScottsdale, Arizona; 4 Paradise Valley Community CollegePhoenix, Arizona

**Keywords:** Cancer education, cancer survivorship, cancer symposia, educational intervention, patient education, quality of life

## Abstract

To evaluate the impact of a patient-centered symposium as an educational intervention on a broad population of cancer patients. We developed a comprehensive patient symposium. Through voluntary questionnaires, we studied the impact of this cancer symposium on quality of life, cancer-specific knowledge, and symptom management among cancer patients. Symposium attendees were provided surveys prior to and 3 months following the educational intervention. Surveys included (1) EORTC-QLQ-C30; (2) disease understanding tool developed for this conference; (3) validated disease-specific questionnaires. Changes over time were assessed using McNemar's tests and paired *t*-tests for categorical and continuous variables, respectively. A total of 158 attendees completed the pre-convention survey. Most respondents reported at least “quite a bit” of understanding regarding treatment options, screening modalities, symptomatology, and cancer-related side effects. Attendees endorsed the least understanding of disease-related stress, risk factors, fatigue management, and legal issues related to disease/treatment. At 3 months, there was improvement in understanding (12 of 14 areas of self-reported knowledge especially regarding nutrition, and stress/fatigue management). However, no significant change was seen in QLQ-C30 functioning, fatigue, pain, or insomnia. A patient symposium, as an educational intervention improves a solid knowledge base amongst attendees regarding their disease, increases knowledge in symptom management, but may be insufficient to impact QoL as a single intervention.

## Introduction

Cancer survivorship demonstrates unmet needs and educational deficits. A growing field of research in oncologic care 1 and health care in general 2 is a focus on quality of life and survivorship. Increased survival is balanced with long-term sequela of treatment, chronic symptom burden, recurrence monitoring, and interrelationship struggles 3,4. These issues impact individuals during the acute phase of diagnosis and treatment, but can evolve or persist in the long-term phase of survivorship 5,6. In order to effectively address these issues, patients may require a variety of resources of support and information.

We developed a comprehensive patient symposium (Living with Cancer [LWC]: A Mayo Clinic Symposium for Patients and Loved Ones) with 8 h of general sessions (covering nutrition exercise, cancer inheritance, financial and legal challenges, communication, survivorship, and cancer therapy) and a 4 h disease-specific breakout (myeloid diseases, myeloma, lymphoma, chronic lymphocytic leukemia, breast cancer, lung cancer, cutaneous malignancy, colorectal cancer, and prostate cancer). Through voluntary questionnaires, we studied the impact of this cancer symposium on quality of life, cancer-specific knowledge, and symptom management among cancer patients.

To date no studies have evaluated the effect of a cancer symposium as an educational intervention to improve quality of life among a general population of cancer patients also provided disease-specific sessions. The aim of this study was to evaluate the impact on quality of life, disease-specific knowledge base, individual function, and symptom burden and management of a patient-centered cancer symposium on a broad population of cancer patients to identify areas of educational and management deficit.

## Methods

### Symposium

The patient symposium was comprised of a 2-day educational conference including 8 h of general cancer education sessions (topics: nutrition, exercise, cancer inheritance, financial and legal challenges, communication, survivorship, and cancer-specific therapy) with presenters from medical oncology, pharmacy, nursing, complementary, and integrative medicine (see Table 1). Additionally, a 4 h disease-specific breakout session allowed individuals to participate in disease-specific education (diagnosis, treatment, future directions), as well as, a question-and-answer session with physicians specialized in the particular disease (breast cancer, chronic lymphocytic leukemia, colorectal cancer, cutaneous malignancy, lung cancer, lymphoma, myeloma, myeloid disease, and prostate cancer) (see Table 1).

**Table 1:** Living with cancer symposium topics and utilized in the pre- and post-intervention survey

Study population	Topic	Questionnaires
All participants	Radiation therapy	European
	Cancer surgery	Organisation for Research and Treatment of Cancer Quality of Life Questionnaire (EORTC)
	Targeted cancer care	
	Alternative medicine in cancer	
	Nutrition	
	Chemotherapy	
	Prescription medicine	
	Self help: energy and fatigue	EORTC QLQ-C30
	Legal and insurance issues	Disease-specific questionnaire
	Relaxation techniques	
	Ethnicity in cancer	
	Communication	
	Cancer survivorship	
	Spirituality and cancer	
	Cancer journey lessons	
	Arts and healing	
Small group breakout session	Acute leukemias	Functional Assessment of Cancer Therapy (FACT)
		FACT-Leuk (Leukemia)
		Myeloproliferative Neoplasm Symptom Assessment Form (MPN-SAF)
	Myeloproliferative disorders/myelodysplastic syndrome	MPN-SAF
	Myeloma/amyloid	EORTC-My (myeloma)
	Chronic lymphocytic leukemia	FACT-Lym (Lymphoma)
	Lymphoma	FACT-Lym (lymphoma)
	Breast cancer	EORTC QLQ-BR23 (breast cancer)
	Lung cancer	EORTC QLQ-LC13 (lung cancer)
	Prostate cancer	EORTC QLQ-PR25 (prostate cancer)
	Melanoma	FACT-M (melanoma)
	Head and neck	EORTC QLQ-H&N35 (head and neck cancer)
	Colorectal cancer	EORTC QLQ-CR29 (colon cancer)

EORTC, European Organisation for Research and Treatment of Cancer Quality of Life Questionnaire; FACT, Functional Assessment of Cancer Therapy; MPN-SAF, Myeloproliferative Neoplasm Symptom Assessment Form.

### Patient selection

Surveys were distributed to attendees of the Mayo Clinic “Living with Cancer” patient symposium prior to their arrival in January 2013. While many attendees were accompanied by familial or friend support only individuals diagnosed with a malignancy, currently or previously, were asked to return the questionnaires.

### Data measurement

Surveys (Table1) included the European Organisation for Research and Treatment of Cancer Quality of Life Questionnaire (EORTC QLQ-C30) which all attendees regardless of cancer diagnosis were instructed to complete. A set of questions (Table2) pertaining to understanding of disease (assessed using response options of not at all, a little, quite a bit, and very much) was developed specifically for this conference.

**Table 2:** Disease-specific questionnaire and percentage of respondents completing both surveys (*N* = 115) who reported understanding [Table-fn tf2-1]of their disease pre- and post-convention

I understand…	Pre-convention (%)	Post-convention (%)	*P*-value
My disease	84	89	0.26
Risk factors that can lead to my disease	48	58	0.11
Screening tests for my disease	88	89	1.0
Symptoms associated with my disease	75	75	1.0
Treatment options for my disease	85	82	0.52
Treatment side effects of my disease	70	75	0.42
What to eat for my health	67	86	<0.001
How to manage disease-related stress	48	65	<0.001
How to manage disease-related fatigue	44	64	<0.001
Legal issues of my disease and treatment	29	36	0.16
The process of making end of life decisions	61	69	0.23
How to maintain my relationships	61	75	0.009
How to navigate the health-care system	55	60	0.39
How to manage financial considerations	53	61	0.14

“Quite a bit” or “very much” response on Cancer Knowledge Questionnaire.

In addition to the EORTC QLQ-C30 and symposium-specific questionnaires all participants completed, attendees completed validated disease-specific questionnaires based on cancer type (see Table1). These questionnaires included the European Organization for Research and Treatment of Cancer Quality of Life Questionnaire for Breast Cancer (EORTC QLQ-BR23), European Organisation for Research and Treatment of Cancer Quality of Life Questionnaire for Colorectal Cancer (EORTC QLQ-CR29), European Organisation for Research and Treatment of Cancer Quality of Life Questionnaire for Head and Neck Cancer (EORTC QLQ-H&N35), European Organisation for Research and Treatment of Cancer Quality of Life Questionnaire for Lung Cancer (EORTC QLQ-LC13), European Organisation for Research and Treatment of Cancer Quality of Life Questionnaire for Myeloma (EORTC QLQ-MY20), European Organisation for Research and Treatment of Cancer Quality of Life Questionnaire for Prostate Cancer (EORTC QLQ-PR25), Myeloproliferative Neoplasm Symptom Assessment Form (MPN-SAF) the Functional Assessment of Cancer Therapy for Leukemia (FACT-Leu), Functional Assessment of Cancer Therapy for Lymphoma (FACT-Lym), and the Functional Assessment of Cancer Therapy for Melanoma (FACT-M).

### Data collection

Patients completed this set of questionnaires prior to completion of the symposium. At 3 months, the patients who completed the initial set of questionnaires were sent a follow-up questionnaire comprised again of the EORTC-QLQ-C30, disease-specific knowledge, and disease-specific validated questionnaires.

### Data analysis

This study was approved by Mayo Clinic Arizona Institutional Review Board. Changes over time were assessed using McNemar's tests and paired *t*-tests for categorical and continuous variables, respectively.

## Results

### Patients

**Figure 1:**
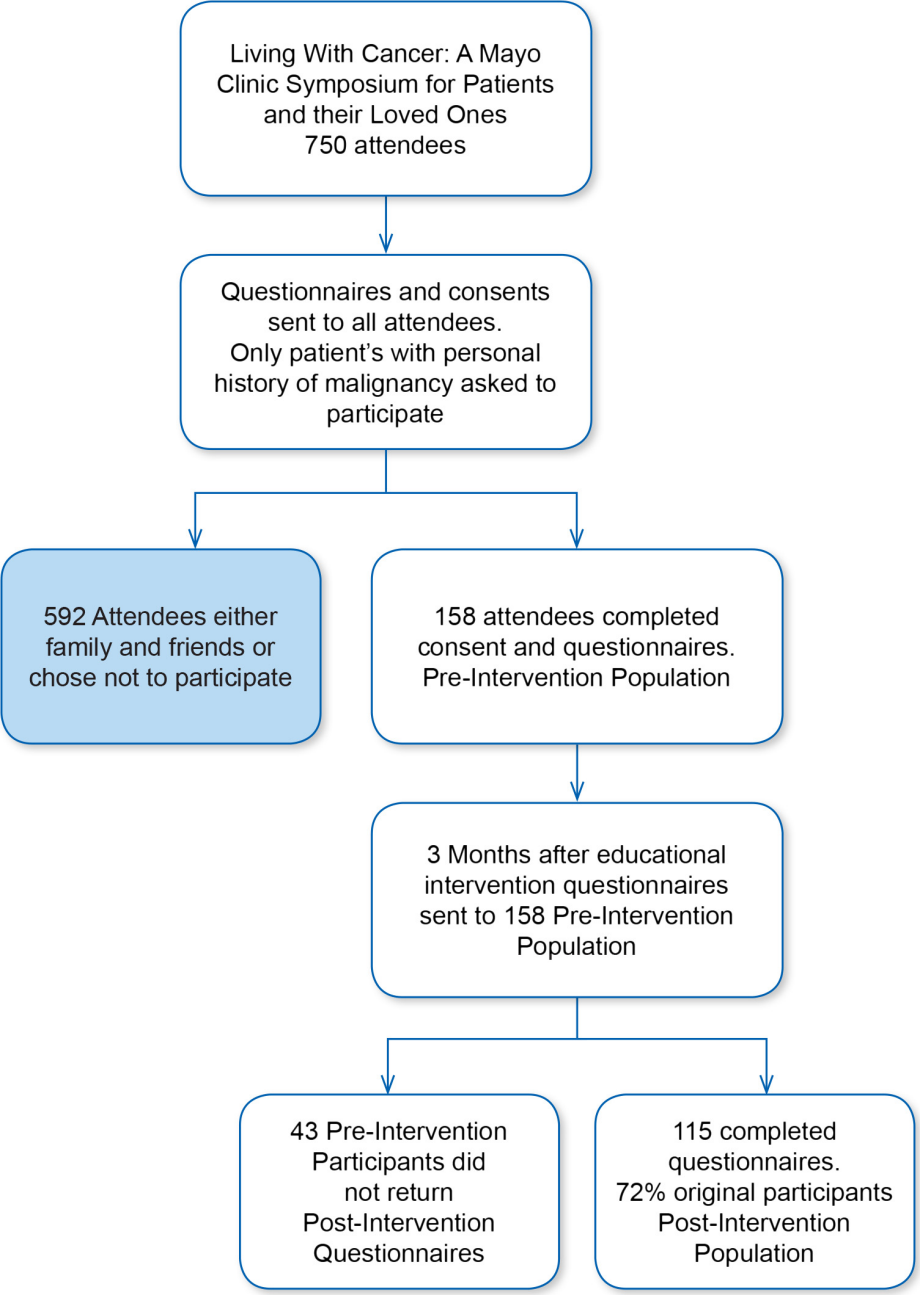
Flow diagram of participant enrollment.

A total of 158 attendees completed the pre-convention survey and 115 of those completed the post-convention survey (see Fig.1).

**Figure 2:**
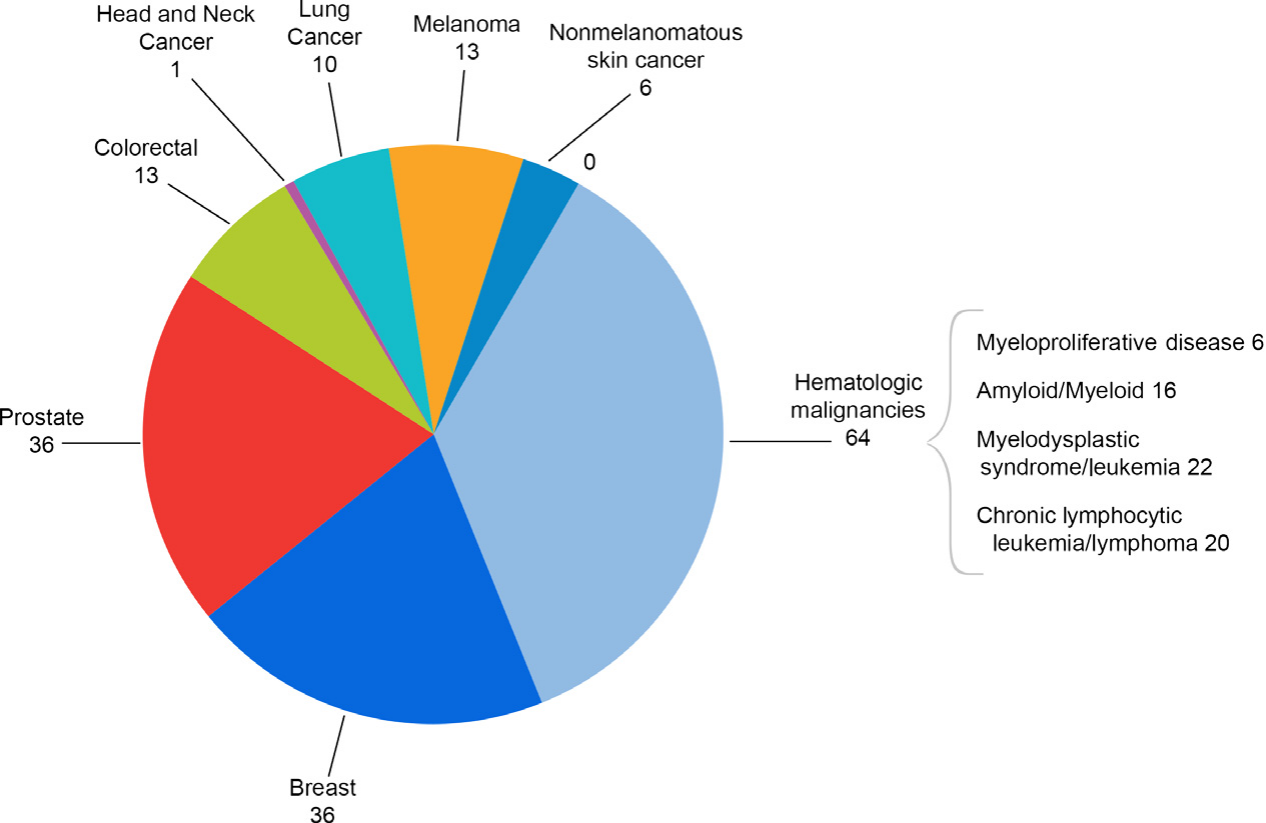
Number of specific cancer diagnoses reported in pre-intervention participants. *N* = 158 individuals.

**Figure 3:**
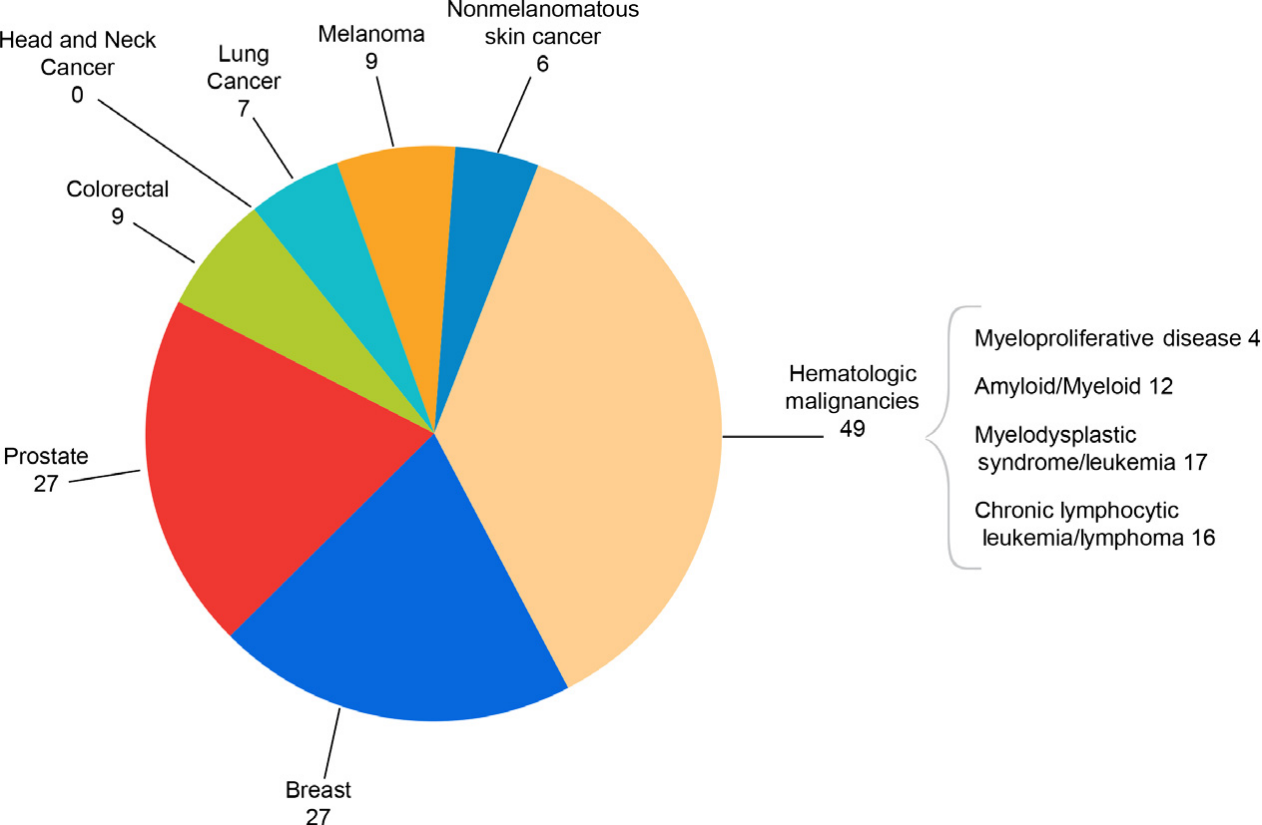
number of specific cancer diagnoses reported in post-intervention participants. *N* = 115 individuals.

The median age was 67.5 years with a range of 30–86 years. Males and females were equally represented in the pre-survey population (Table3). Cancer disease type included 40% hematologic malignancies (*n* = 64), 23% breast cancer (*n* = 36), 23% prostate cancer (*n* = 36), and 17% other (see Fig. 2 and Fig.3). The majority of respondents were greater than 1 year from cancer diagnosis (76%), with 40% greater than 3 years from diagnosis. Most participants had undergone treatment of some sort by the time of the symposium.

**Table 3:** Demographic data

Demographic	Pre-intervention participants	Post-intervention participants
Number of participants	155	115
Age (years, mean)	67.5	68.3
Male gender (%)	77 (49)	55 (48)
Time since diagnosis >1 year	76%	74%
Previous types of treatment		
Surgery	96 (61%)	72 (63%)
Chemotherapy	76 (48)	54 (47)
Radiation	58 (37)	42 (37)
Hormonal therapy	23 (15)	19 (17)

### Baseline disease understanding, symptoms, QOL

The study revealed participation by sophisticated patients. Most attendees endorsed understanding their disease quite a bit (54%) or very much (29%). This sufficient level of understanding, greater than 65% of respondents reporting quite a bit or very much was seen regarding disease-specific treatment options (85%), screening modalities (88%), symptomatology (75%), cancer-related side effects (70%), and nutrition (67%). Additionally, a subset of disease-specific topics revealed a modest knowledge level, between 50% and 65% respondents reporting quite a bit or very much understanding. The topics of modest knowledge included end of life decision making (61%), relationship skills (61%), health-care navigation (55%), and financial management (53%). While disease-specific knowledge was fair for most topics assessed, there were distinct knowledge gaps identified. Attendees endorsed the least understanding of disease-related stress (48%), risk factors (48%), management of disease-related fatigue (44%). The least understanding was related to legal issues of disease/treatment (29%).

One-fifty-eight respondents completed the EORTC QLQ-C30 with mean scores of 84.4 (SD = 16.6) for physical functioning, 83 (SD = 22.3) for role functioning, 80.7 (SD = 22.9) for social functioning, and 72.7 (SD = 20.2) for global health status. The three symptoms with highest mean scores were insomnia (mean = 35.5, SD = 32.4), fatigue (mean = 29.3, SD = 22.4), and pain (mean = 22.7, SD = 24.7).

### Post course assessment at 3 months

One hundred and fifteen patients (72.8% of respondents) who completed the pre-symposium survey also completed the post-symposium survey. Those who completed both surveys had a mean age of 68.3, with both men and women still equally represented (47.8% men, 52.2% women).

### Knowledge improvement durable at 3 months

There was improvement in 12 of 14 areas of self-reported knowledge (refer to Table 2) especially in areas of nutrition, and management of disease-related stress and fatigue (all *P* < 0.001). There was a statistically significant improvement in understanding the post symposium setting regarding nutrition, with 86% reporting a good (quite a bit/very much), increased from 67% prior to the symposium (*P* < 0.001). Of the four topics with moderate understanding in the pre-intervention period, two of these topics demonstrated enough improvement to transition to sufficient understanding, with greater than 65% of respondents in the post-convention assessment indicating very much or quite a bit of understanding of making end of life decisions (69% post, 61% pre) and relationship skills (75% post, 61% pre). Despite improvement in knowledge of health-care navigation and finance management, these two topics persisted in only the moderate level of knowledge. Of the four topics with the least level of knowledge in the pre-convention data, three of the four improved to moderate knowledge: stress management (65% post, 48% pre, *P* < 0.001), fatigue management (64% post, 44% pre, *P* < 0.001), and disease risk factors (58% post, 48% pre). The most significant knowledge deficit in the pre-convention participants persisted post-intervention despite some improvement regarding legal issues of disease and treatment (36% post, 29% pre).

### Symptomatic changes at 3 months

The EORTC QLQ-C30 post symposium data revealed no significant change in physical functioning (mean score 84.8 vs. 84.9 post symposium, *P* = 0.9), role functioning (84.5 vs. 84.3, *P* = 0.93), emotional functioning (79.8 vs. 79.4, *P* = 0.8), fatigue (28.3 before vs. 29.1 after, *P* = 0.65), pain (18.6 before vs. 17.5 after, *P* = 0.82), or insomnia (31 before vs. 29.6 after, *P* = 0.58). The only statistically significant change was for a slight decrease in global health status or quality of life rating (74.3 before vs. 71.7 after, *P* = 0.02) though the fact that this is clinically significant this is unlikely due to small sample size.

## Discussion

This study revealed several important findings that can inform and help define cancer patient's knowledge deficits and needs. The knowledge level of patients diagnosed with cancer choosing to participate in a cancer symposium is good, with the majority of patient's reporting a fair understanding of disease-specific topics. The strongest levels of knowledge were primarily diagnostic and treatment-related (screening tests, symptoms of disease, treatment options, and side effects) which may be the most likely topics covered when encountering the health-care system. The topics of symptom management (stress, fatigue), health-care logistics (navigation, legal issues, end-of-life decision making, financial concerns), interpersonal skills (relationship management) were each less confidently reported by convention attendees. These topics may be less universally discussed during health-care encounters with providers, which may contribute to the information deficits. Importantly, many of these knowledge deficits were able to improve, some of statistical significance, merely by attending a 2-day patient-centered symposium.

There is obvious patient interest for acquiring knowledge on cancer in various stages of diagnosis and treatment, evidenced by attendance at this patient symposium. This fact is well documented in the literature. Patients diagnosed with cancer experience a spectrum of psychosocial, physical, emotional and financial burdens. Patients with diagnosis of cancer and their families experience information need and knowledge deficit 7,8. Education deficit may contribute to impaired quality of life, adding to anxiety, symptom mismanagement, and relationship struggles. This knowledge deficit may be at diagnosis, treatment, or in the posttreatment period 9. Improvements in cancer diagnosis and treatment have improved some cancer outcomes, but many aspects of cancer care and survivorship remain underdiagnosed leading to impaired quality of life 10.

It is not merely attendance that demonstrates patient interest, but questionnaire completion rates suggest that patients interested in improving their cancer knowledge are active in seeking and participating in the learning process. Providing information to patients with cancer can improve patient experience. For example, providing information to patients can reduce patient reported distress 11,12 and anxiety 13. Additionally, patient education can improve patient experience with decision making 8,14.

Education interventions may serve to enable improved symptom control. The main symptoms reported by LWC participants were pain, insomnia, and fatigue. These symptoms are commonly reported in cancer patients, both actively undergoing treatment and in the posttreatment setting. Education can be provided through various avenues to address patient pain. For example, the Expert Patient Program in the United Kingdom based on 6 weeks of teaching individuals with cancer self-care techniques including action planning, relaxation, fitness, communication, nutrition. Evaluation of the program's efficacy revealed patient participants reported improved physical exercise, communication, health distress, and energy 15.

While a patient-centered symposium has not been evaluated in the cancer literature, formalized programs based on instruction and information provision have been studied in cancer patients. A self-care education program based on group discussion and direct patient instruction in esophageal cancer patients who were scheduled to receive chemotherapy in the upcoming month regarding disease knowledge, expectations for both surgery and chemotherapy side effects, diet regimen, exercise recommendation, pain control techniques, and fatigue and sleep discussions revealed that the intervention group demonstrated improved quality of life parameters of physical, emotion, role and social functioning and decreased symptoms of nausea, vomiting, pain, dyspnea, insomnia, and weight loss when compared to controls 16.

Another study, concluded that a 12-week Internet-based education program focused on energy conservation, physical activity, sleep, pain control, and stress management demonstrated decrease in fatigue, anxiety, and improvement in emotional functioning in cancer patients who reported moderate or great fatigue at baseline 17.

Additionally, a systematic review of four randomized controlled trials evaluating the efficacy of orientation programs in cancer patients newly was diagnosed. The study showed the orientation programs demonstrated increased knowledge regarding cancer in general and treatment side effects, improved patient reported anxiety, distress, depressive symptom burden. However, the orientation programs were heterogenous and did not measure harms, and the concern for level of evidence led to inconclusive support for these programs, including at what time in the process of diagnosis and treatment these programs are the most effective 18.

While the most common troublesome symptoms reported at the conference (pain, insomnia, fatigue) are well known to the cancer symptom literature, the patient knowledge gaps identified are less well identified in the literature. Cancer patients demonstrate significant knowledge needs and information deficits throughout their health-care experience, from diagnosis, through treatment, and post -treatment in the survivor stage 9. A review of 19,000 phone calls from cancer patients to the National Cancer Institute's Cancer Information Service between September 2002 and August 2003 revealed 40% of patients calling for information were actively undergoing cancer-directed treatment, 20% were in a posttreatment phase, 68% were female, and nearly 50% were over the age of 60. Sixty percent of calls requested cancer-specific treatment information, 32% general cancer site information, 15% support services, 7% psychosocial issues, and 5% requested cancer screening and diagnosis information. Male callers were more likely to request specific treatment information compared to female callers, while female callers were more likely to request information on support services, psychosocial issues, and cancer screening and diagnosis. The participants in this study demonstrate a baseline knowledge level regarding their cancer likely more robust than the general cancer patient population, as this particular group selected to attend this symposium. That being said, there were knowledge deficits even in this patient population.

Interestingly, the knowledge gaps of stress management and legal issues demonstrate new areas of focus for health-care providers to help address patient needs. Legal knowledge was a least endorsed topic in the pre- and post intervention questionnaires and did not improve significantly despite a session devoted to the topic at the conference. Patient-centered assessment or delivery of legal education is scarcely reported in the cancer literature, if at all. This may be that health-care providers are unaware of the legal issues patients may experience during or after cancer diagnosis, time is limited during interaction with health-care providers with appointments spent on medical aspects of treatment and monitoring, or that providers may feel ill-equipped to discuss legal issues due to lack of knowledge themselves.

The intervention improved knowledge level more significantly than quality of life. This symposium benefit is likely multifactorial. The symposium was designed to maximize dissemination of information covering a variety of topics of cancer therapy, management, and survival. Not surprisingly, baseline knowledge was improved by attending a symposium with this aim. Additionally, as the study population was comprised of various ages, cancer diagnoses, time since diagnosis or treatment, the participants are not a homogenous group. The quality of life of individuals diagnosed with cancer can evolve over time depending on treatment, interactions with the health-care system, development, or resolution of symptoms, or impact on social and physical functioning, and the homogenous group of participants likely experienced the same evolution of cancer care. This suggests that participants were at different locations along the continuum of cancer care, and thus a quality of life assessment at two points in time could identify some individuals surveyed with a reported increase or decrease in quality of life due to the chronicity of their disease.

## Conclusions

The patient-centered cancer symposium provided a unique opportunity to assess QOL and disease understanding in response to an educational intervention. Cancer patients endorsed a high baseline understanding of their disease and appeared motivated for an educational teaching program. Respondents reported a high level of insomnia with other symptoms and QOL domains being consistent with a general cancer population. Despite the hope that quality of life could improve with attending the symposium, this was not the case. It may be possible, that with better identification of knowledge deficit, needs, and provision, that quality of life may be improved for attendees but further research is required.

This first assessment of a patient-centered cancer symposium as an education intervention provides several important observations. First, patients self-selecting to participate in such an event seek to improve an already solid knowledge base regarding their malignant disease. Second, participating in the event strongly improved both disease-specific knowledge and most strongly aided the pre course deficit in issues of managing the illness (financial, legal, etc.) and survivorship. Third, as a single intervention the convention did not improve specific symptoms with participation. Future efforts on (1) improving participation with more disease knowledge “naïve” patients and (2) targeted efforts aimed at specific symptom targeting (i.e., insomnia, stress, fatigue) are planned.

Future studies should evaluate cancer symposium as a possible intervention modality to affect patient knowledge, desired knowledge among patients with hematologic and solid tumor malignancies.

## Conflict of Interest

None declared.
